# Whose Prosocial Intentions Are More Affected by Mindfulness, Young Adolescents or Young Adults?

**DOI:** 10.1002/pchj.70036

**Published:** 2025-07-06

**Authors:** Qianguo Xiao, Chenyu Li, Chen Chen, Jialan Ma

**Affiliations:** ^1^ School of Management Zunyi Medical University Zunyi China; ^2^ Department of Psychology Wuhan Sports University Wuhan China; ^3^ Department of Psychology Inner Mongolia Normal University Huhehaote China

**Keywords:** mindfulness, moral disengagement, moral identity, prosocial willingness

## Abstract

Two studies were conducted to investigate: (1) the effects of dispositional mindfulness and short‐term mindfulness induction on prosocial willingness, (2) the mediating roles of moral identity and moral disengagement, and (3) age‐related differences between young adolescents (12–15 years) and young adults (18–24 years). In Study 1, a cross‐sectional survey was conducted among 271 college students (young adults) and 229 middle school students (young adolescents), assessing dispositional mindfulness, moral identity, moral disengagement, and prosocial willingness. In Study 2, an experimental design was employed to explore the short‐term effects of two types of mindfulness inductions (with ethical elements or without) on these variables, involving 105 young adults and 142 young adolescents. Study 1 revealed that, in adolescents, moral identity significantly mediated the relationship between dispositional mindfulness and prosocial willingness, while moral disengagement served as the primary mediator among adults. Study 2 showed that different short‐term mindfulness inductions significantly affected moral identity, moral disengagement, and prosocial willingness in adolescents, with significant mediation effects of moral identity and moral disengagement. However, these effects were not significant in adults. Both types of mindfulness induction showed differential mediating effects, suggesting age‐specific psychological mechanisms. Findings highlighted age‐related differences in how mindfulness influences prosocial behavior, mediated by moral constructs. Both studies consistently showed that, for adolescents, the moral psychology (such as moral identity and moral disengagement) significantly influences the association between mindfulness (interventions) and prosocial behavior. This provides important insights into ethical mindfulness education, emphasizing the need to account for psychological development characteristics when designing mindfulness programs for adolescents.

## 1 Introduction

Mindfulness refers to maintaining a moment‐by‐moment awareness of our thoughts, feelings, bodily sensations, and surrounding environment intentionally (Kabat‐Zinn 2003). Over the past decade, the intersection between mindfulness practice and prosocial behavior has become a prominent research focus, particularly concerning the role of dispositional mindfulness and mindfulness interventions in promoting prosocial behaviors.

However, existing studies reveal inconsistencies, often influenced by factors such as individual differences and social contexts (Iwamoto et al. 2020; Schindler and Friese 2022). While numerous studies suggest a positive correlation between dispositional mindfulness or mindfulness interventions and prosocial behaviors in adult populations (Berry et al. 2018; Hafenbrack et al. 2020), the relationship between mindfulness and prosociality is not straightforward (Malin 2023).

Some studies suggest that mindfulness meditation can inhibit prosocial behavior, particularly among individuals with higher independent self‐construals, with age differences potentially moderating this effect. The role of moral identity (MI) and moral disengagement (MD) further complicates these findings (Poulin et al. 2021; Malin 2023). Therefore, when examining the effects of mindfulness (intervention) on prosocial behaviors, it is crucial to consider significant individual differences, particularly age‐related variations in MI and MD.

### 1.1 The Age‐Related Difference in MI and MD

Age‐related developmental differences in MI and MD are critical to understanding prosocial behavior. MI refers to the degree to which an individual self‐identifies with moral traits, which involves internalizing moral values and viewing them as central to one's self‐concept (Cheryan and Bodenhausen 2000; Aquino and Reed 2002). It is an important motivational factor that influences one's moral actions (Hardy and Carlo 2011). Several studies indicate that significant age‐related differences exist in the development of MI (Doering 2013; Krettenauer and Victor 2017). For instance, Krettenauer et al. (2016) found that cross‐context differentiation in MI increases during adolescence but declines with age as individuals transition into adulthood. This phenomenon may be linked to the overarching developmental characteristics inherent within personality and social psychology. Adolescence is recognized as a tumultuous period, during which individuals tend to exhibit unstable emotions (Kabir 2013) and an increased differentiation of values (Ahn et al. 2022). Decisions and behaviors during this developmental stage are particularly susceptible to external influences, such as peer pressure in educational settings (Benish‐Weisman et al. 2022; Cooley et al. 2019). As individuals progress from adolescence into adulthood, their personalities tend to stabilize and mature (Roberts and Wood 2006); this maturation elevates the importance of the moral qualities defining moral personality. These developmental differences contribute to the evolution of individual MI from adolescence to adulthood, characterized by a shift from concrete to abstract thinking, from extrinsic motivation to intrinsic motivation, and from a prevention orientation to a promotion orientation (Krettenauer 2022). These age‐related differences in MI are also related to the difference in MD (Detert et al. 2008).

MD, according to Bandura (2016) and Schaefer and Bouwmeester (2021), refers to cognitive mechanisms that allow individuals to engage in immoral behavior without feeling distress through reconstructing moral judgments through mechanisms of moral reasoning. Generally, individuals exhibiting a greater tendency toward MD are more likely to demonstrate lower intentions to engage in fewer helping behaviors and display increased antisocial behaviors (Atkinson et al. 2022). Research indicates that significant age‐related differences also exist in MD. For instance, some longitudinal studies have shown that individuals in early and middle adolescence, particularly those aged 14–16, are more prone to MD than their peers in other age groups (Caroli and Sagone 2014; Gini et al. 2014; Paciello et al. 2008). In the case of young adults, it is generally posited that, concurrent with the maturation of self‐regulation abilities and an increasing emphasis on social roles, MD tends to decrease with age. However, this difference may be associated with social contexts. One longitudinal study from Spain found that older students and men tend to engage in more MD than younger students and women (Romera et al. 2021). Some cross‐sectional meta‐analyses and longitudinal studies from China have shown that MD among Chinese college students exhibits a decreasing trend over time (Jin et al. 2023; Xiangkun et al. 2024). Overall, the literature indicates significant differences between adolescents and adults in MD and its influencing factors (Volk et al. 2015).

### 1.2 The Associations of Mindfulness, MI, MD, and Prosociality in Age‐Related Differences

From both traditional mindfulness practices and modern scientific research, there is a positive correlation between mindfulness (practice) and moral cognition. Traditionally, mindfulness practice places great emphasis on moral ethics, such as the cultivation of compassion and altruism. From the perspective of modern scientific research, numerous studies have shown that mindfulness is positively related to moral psychology and behavior, although the effect sizes vary (Donald et al. 2019). Some scholars have further proposed an experimental and theoretical framework to explain the positive moral or prosocial effects of mindfulness practice, arguing that heightened awareness of physiological and mental phenomena through mindfulness meditation may lead to enhanced detection of morally relevant internal and external cues and may thereby foster the emergence of moral behaviors (Sevinc and Lazar 2019). However, as mentioned earlier, the relationship between mindfulness and moral cognition (including prosociality) is not straightforward due to the age‐related differences, such as differences in MI and MD. Then, we are more concerned with the age‐related differences (in young adolescents and young adults) between mindfulness (intervention), MI, MD, and prosociality.

#### 1.2.1 The Associations of Mindfulness, MI and Prosociality in Age‐Related Differences

For young adults (e.g., college students aged 18–24 years), the mediating role of MI between dispositional mindfulness and prosociality may be negligible, so it may be difficult for mindfulness interventions to modify these relationships. There are two main theoretical hypotheses as to why mindfulness (intervention) is positively associated with prosocial behavior: it helps to be aware of the needs of others, and it may help to improve the moral sensitivity or empathy of the self (Schindler and Friese 2022), which were supported by some cross‐sectional studies showing that mindfulness and prosociality are associated with empathy (Guo et al. 2022), self‐identity, and self‐perspective (Atkins and Styles 2015). The developmental characteristics of MI and MD may not guarantee the association between mindfulness and prosociality in young adults. Research on the development of MI indicates that external MI motivation generally diminishes with age, while internal MI motivation tends to increase, where a higher degree of internalization of MI correlates with reduced participation in donation behaviors and public engagement (Gotowiec and van Mastrigt 2019). Schindler and Pfattheicher (2021) found no evidence supporting a positive correlation between trait mindfulness and actual charitable donations, nor participation in incentivized economic games. From the perspective of intervention, mindfulness intervention, especially short‐term mindfulness practice, may also be difficult to improve the MI, MD, and prosociality of young people. For instance, Xie et al. (2023) suggested that 8‐week mindfulness training enhanced participants' mindfulness levels but reduced willingness to help in low‐cost situations. Xiao et al. (2020) also suggested that an 11‐week mindfulness intervention just significantly improved the willingness to engage in prosocial behavior among college students with higher MI. Consequently, among young adults, a positive association between mindfulness (intervention) and prosocial behavior, or the willingness to engage in such behavior, may not be evident.

However, this assertion may not apply to young adolescents (e.g., middle school students aged 12–15 years). For this demographic, MI serves as a crucial self‐regulatory mechanism encouraging the maintenance of a moral self‐image (Xu et al. 2023). According to Kohlberg's stage theory of moral development and recent studies, the moral development of adolescents aged 9–15 is characterized by a recognition‐seeking orientation and adherence to rules and order, with the symbolic or external component of their MI occupying a dominant position at this stage (Krettenauer 2020). Children within this demographic often define MI through their desire to meet the expectations of their moral community and pursue self‐presentational goals or self‐affirmation (Jennings et al. 2015). Consequently, young adolescents with high levels of symbolic MI tend to exhibit greater prosocial behaviors to demonstrate their moral characteristics to others. Furthermore, MI and prosocial behavior among adolescents are more susceptible to educational interventions, including mindfulness inductions.

#### 1.2.2 The Associations of Mindfulness, MD and Prosociality in Age‐Related Differences

Similarly, when examining the relations among mindfulness (intervention), MD, and prosociality, notable differences may exist between adolescents and adults. Research indicates that during the self‐integration period, young adolescents exhibit greater variability in MD and are more susceptible to external factors, such as school climate and peer influence (Wang et al. 2019). For example, a study employing MD‐based situational tests across grades 5–8 found fluctuating differences in MD among various grades (Gao and Zhang 2017). Specifically, they discovered that MD was significantly higher in grade 5 than in other grades, while seventh‐grade students exhibited significantly lower levels of moral justification, blame attribution, and advantage comparison compared to their peers. This finding suggests that the prevailing symbolic or external aspects of young adolescents' MI may lead them to downplay MD to preserve their self‐image. They tend to appraise themselves higher on MI while rating lower on MD (Hardy et al. 2015). Consequently, there may be a negative association between mindfulness (intervention) and MD and the willingness to engage in prosocial behaviors for young adolescents. Some research also provides indirect support. For example, Bussey and Luo (2024) suggested that mindfulness moderated the association between moral disengagement and cyberbullying perpetration for youth aged between 10 and 16 years significantly. Georgiou et al. (2020) found that, for youth aged 14 to 17 years, the association among mindfulness, bullying, and victimization was fully mediated by moral disengagement (Georgiou et al. 2020).

In contrast, the situation appears distinct for young adults. According to the preceding analysis, the transition from adolescence to young adulthood is associated with a more consistent and internalized MI, which may not necessarily align with higher levels of prosocial behavior (Hardy et al. 2015). Although some studies indicate that MD generally declines with age, in alignment with the internalization of self‐identity and the maturation and stabilization of values (Milfont et al. 2016), young adults may exhibit a relatively stable pattern of MD. For example, a 12‐year longitudinal panel study indicated that young adults exhibited the greatest stability in values related to stimulation, conformity, and universalism (Leijen et al. 2022). What's more interesting, studies have also found that mindfulness significantly decreases MD and consequently curtails workplace unethical behavior predominantly among individuals with low moral identity (Ming et al. 2025). These studies suggest that stable or high levels of MD may be aspects of individuals' underlying (undesirable) moral reasoning patterns and values that lead to more stable and consistent (unethical) behavior. Therefore, we infer that for adults, MD may have a significant mediating effect between mindfulness and prosocial behavior intention, but short‐term mindfulness intervention may be difficult to affect MD and prosocial behavior intention.

#### 1.2.3 The Associations Between Mindfulness Interventions and Prosociality

In addition to age‐related differences, the complex and unstable relationship between mindfulness interventions and prosociality may also be related to the type and duration of mindfulness interventions. For example, whether the mindfulness intervention includes moral and ethical elements such as compassion. A previous study demonstrated that integrating ethical elements, such as instructions encouraging participants to recognize the shared humanity of all individuals, into an 8‐day program (referred to as ethical mindfulness) significantly increased the likelihood of donations compared to a generic mindfulness approach (called secular mindfulness). This effect was particularly pronounced among participants under the age of 25 and those with lower educational attainment (Chen and Jordan 2018). Ethical mindfulness, as opposed to traditional didactic methods of moral education, promotes the teaching of values and attitudes more gently and compassionately. Consequently, ethical mindfulness interventions, with their implicit components of moral education, may present an effective avenue for moral development (Meindl et al. 2017), thereby significantly influencing the moral psychology and prosocial behaviors of adolescents, including their willingness to donate. Based on a review of studies discussing the developmental differences in MI between young adolescents and young adults that were articulated in the introduction, we propose that short‐term mindfulness induction, particularly incorporating ethical mindfulness, may exert a substantial influence on the moral psychology and behavioral inclinations of young adolescents but not of young adults.

Based on the above analysis, we hypothesize that there are different pathways of effect among mindfulness (intervention), MI, MD, and prosocial intentions between the two groups of young adolescents and young adults. However, few comparative studies have investigated the distinct roles of MI and MD in mediating the relationships between mindfulness and prosocial intentions among adolescent and adult populations. To address this gap, the current study conducts two comparative studies. Study 1 is a cross‐sectional study, aiming to explore whether there were different mediation mechanisms of MI and MD between mindfulness and prosocial behavior willingness for young adolescents (aged 12–15) and young adults (aged 18–24 years). Study 2 is a single short‐term intervention study to test whether two different short‐term mindfulness inductions (with ethical elements or not) have different effects on the MI, MD, prosocial behavior willingness, and their relationship for the two different groups.

## 2 Study 1

### 2.1 Methods

#### 2.1.1 Participants

A total of 271 undergraduate college students (89 male) and 229 middle school students (119 male) participated in a mental health education research project. All participants voluntarily participated in the survey and were well‐informed. Seventeen participants did not complete the questionnaires; therefore, their data were excluded from the final analysis. The valid response rate was 96.7% (*N* = 271 college students, *M*_age_ ± SD = 19.97 ± 1.21; *N* = 229 middle school students, *M*_age_ ± SD = 13.82 ± 0.90).

### 2.2 Measures

#### 2.2.1 Moral Identity

Investigating MI in the Chinese context needs to map to Chinese people's perspective of being a moral person (Xu and Ma 2014). Therefore, the adolescent version of the MI Questionnaire in Chinese (A‐MIQ) was used to measure the MI of young adolescents. A‐MIQ is adapted by Li (2013) based on the scale initially developed by Aquino and Reed (2002). A‐MIQ is a 24‐item self‐report questionnaire that encompasses two internalization and symbolization dimensions, and each dimension has 12 items. Items are rated on a 5‐point scale ranging from 1 (*strong inconformity*) to 5 (*strong conformity*). The Cronbach's alpha was 0.89 in the present study. The emerging adult version of the MI Questionnaire (E‐MIQ) was adapted by Xu and Ma (2014) based on the scale created by Aquino and Reed (2002) and has been validated as a reliable instrument for measuring MI in the Chinese context. E‐MIQ has two components of internalization and symbolization aspects of MI. Each dimension has five items and a total of 10 items. Items are also rated on a 5‐point scale ranging from 1 (*strong inconformity*) to 5 (*strong conformity*). The higher the score represents the higher the level of MI. The Cronbach's alpha was 0.69 in the present study. Although the two versions of the questionnaire have differences in the number of items, they have the same structure, and both measure the MI of different groups in the Chinese cultural context from the two dimensions of internalization and symbolization.

#### 2.2.2 Moral Disengagement Scale

The MD Scale was adapted to the Chinese context by Wang and Yang (2010), building upon the scale developed by Bandura. The revised version includes eight factors as the original version. A total of 32 items are rated on a scale of 1 (*strongly disagree*) to 5 (*strongly agree*). A higher score indicates a higher level of MD. The results of several sample tests have shown that the questionnaire has good reliability and validity in a Chinese cultural background (Wang and Yang 2010; Xia and Hou 2019). The Cronbach's alpha of this scale in middle school students and college students in this study was 0.84 and 0.93, respectively.

#### 2.2.3 Mindful Attention Awareness Scale (MAAS)

The MAAS has been recognized as a validated tool for measuring mindfulness in both clinical and non‐clinical samples, which assesses individual differences in the frequency of mindful states over time with 15 items. MAAS respondents indicate how frequently they have the experience described in each statement using a 6‐point Likert scale from 1 (*almost always*) to 6 (*almost never*), where high scores reflect more mindfulness. It was tested that the Chinese version of MAAS has acceptable psychometric quality in Chinese young samples (Chen et al. 2012). The Cronbach's alpha of this scale in middle school students and college students in this study was 0.87 and 0.89, respectively.

#### 2.2.4 Donation Scenario

Time, money, or blood donation willingness scenarios are widely used in prosocial studies. Since the scarcity of time may affect donation preferences (Malika et al. 2024), considering that the study time of Chinese middle school students is relatively tight, we designed the following two donation scenarios about books and money to examine subjects' prosocial willingness. The specific scenario topics were as follows: (1) If you have 10 extracurricular books, how many would you be willing to donate any of them to poor students in the mountainous area? (2) If you have 100 RMB extra pocket money, how much would you be willing to donate to poor students in the mountainous area?

#### 2.2.5 Procedure

Data collection was conducted by research team members in middle schools and universities in Guizhou and Chongqing Province using both paper and electronic questionnaires (https://www.wenjuan.com/). Informed consent was obtained from all subjects.

#### 2.2.6 Data Analyses

Descriptive statistics, *t*‐tests, correlation analyses, and ANOVA were performed using SPSS 22.0. For statistical analysis, the PROCESS macro in SPSS was used to test the mediation models.

### 2.3 Results

Table 1 presents descriptive statistics for various variables, including dispositional mindfulness, MI, MD, and the willingness to donate books and money. It also includes the results of the independent samples *t*‐test comparing the young adolescent and young adult groups. The analysis showed no significant differences between the groups regarding their levels of mindfulness or their willingness to donate books or money. In contrast, significant differences were observed in MI (*t* = −11.15, *p <* 0.001, Cohen's *d* = 1.01) and MD (*t* = 21.82, *p <* 0.001, Cohen's *d* = 1.94). Specifically, young adolescents had significantly higher self‐assessed MI scores than young adults (*M* ± SD: 3.69 ± 0.59 vs. 3.15 ± 0.47). In contrast, young adolescents reported significantly lower MD scores (*M* ± SD: 2.93 ± 0.51 vs. 2.03 ± 0.41).

**TABLE 1:** The results of describable statistic and *t*‐test.

	Group (*N*_Young adults_ = 271 *N*_Young adolescents_ = 229)	Skewness	Kurtosis	*M*	SD	*t*
Mindfulness	Young adults	0.056	0.284	3.90	0.69	−0.00
	Young adolescents	−0.244	0.180	3.90	0.85	
Moral identity	Young adults	−0.660	1.887	3.15	0.47	−11.15***
	Young adolescents	−0.587	1.630	3.69	0.59	
	Young adolescents	−0.352	0.671	3.55	0.74	
Moral disengagement	Young adults	−0.091	0.297	2.93	0.51	21.82***
	Young adolescents	0.310	−0.539	2.03	0.41	
Willingness to donate of book	Young adults	−0.704	−0.547	7.10	3.02	1.77
	Young adolescents	−0.611	−0.646	6.62	3.12	
Willingness to donate of money	Young adults	−0.643	−0.701	68.63	32.20	−0.09
	Young adolescents	−0.712	−0.579	68.90	31.27	

*** *p* < 0.001.

### 2.4 The Results of the Correlation Analysis

Table 2 illustrates the correlation coefficients regarding MI, MD, and dispositional mindfulness for both young adolescent and young adult groups. The analysis indicated that, within the young adult group, dispositional mindfulness was negatively correlated with MD (*r* = −0.18, *p <* 0.01), while MI was positively correlated with MD (*r* = 0.19, *p <* 0.01). Furthermore, willingness to donate books exhibited a significant negative correlation with MD (*r* = −0.15, *p <* 0.05). However, dispositional mindfulness did not show a significant relationship with MI. In the young adolescent group, moderate negative correlations were found between dispositional mindfulness and MD (*r* = −0.36, *p* < 0.01) as well as between MI and MD (*r* = −0.35, *p* < 0.01). Additionally, a moderate positive correlation emerged between MI and dispositional mindfulness (*r* = 0.33, *p* < 0.01). These findings, as displayed in Table 2, suggest notable differences in the correlations of these variables between the young adolescent and young adult groups.

**TABLE 2:** Correlations analysis of each variable.

	1	2	3	4	5	6	7
1. Donation willing (book)		0.25**	−0.02	−0.10	−0.10	−0.09	−0.15*
2. Donation willing (money)	0.14*		0.03	0.04	−0.02	0.06	−0.06
3. Mindfulness	0.09	0.01		0.01	−0.06	0.05	−0.18**
4. Moral identity	0.12	0.21**	0.33**		0.76**	0.91**	0.19**
5. Internalization identity	0.08	0.17**	0.28**	0.86**		0.43**	0.21**
6. Symbolization identity	0.13**	0.20**	0.31**	0.92**	0.60**		0.14**
7. Moral disengagement	−0.08	−0.12	−0.36**	−0.35**	−0.26**	−0.35**	

*Note*: The correlation coefficient of the upper right triangle of the matrix is young adults, and the correlation coefficient of the lower left triangle is young adolescents.

** *p* < 0.01.

* *p* < 0.05.

### 2.5 The Results of the Mediation Analysis

**FIGURE 1:**
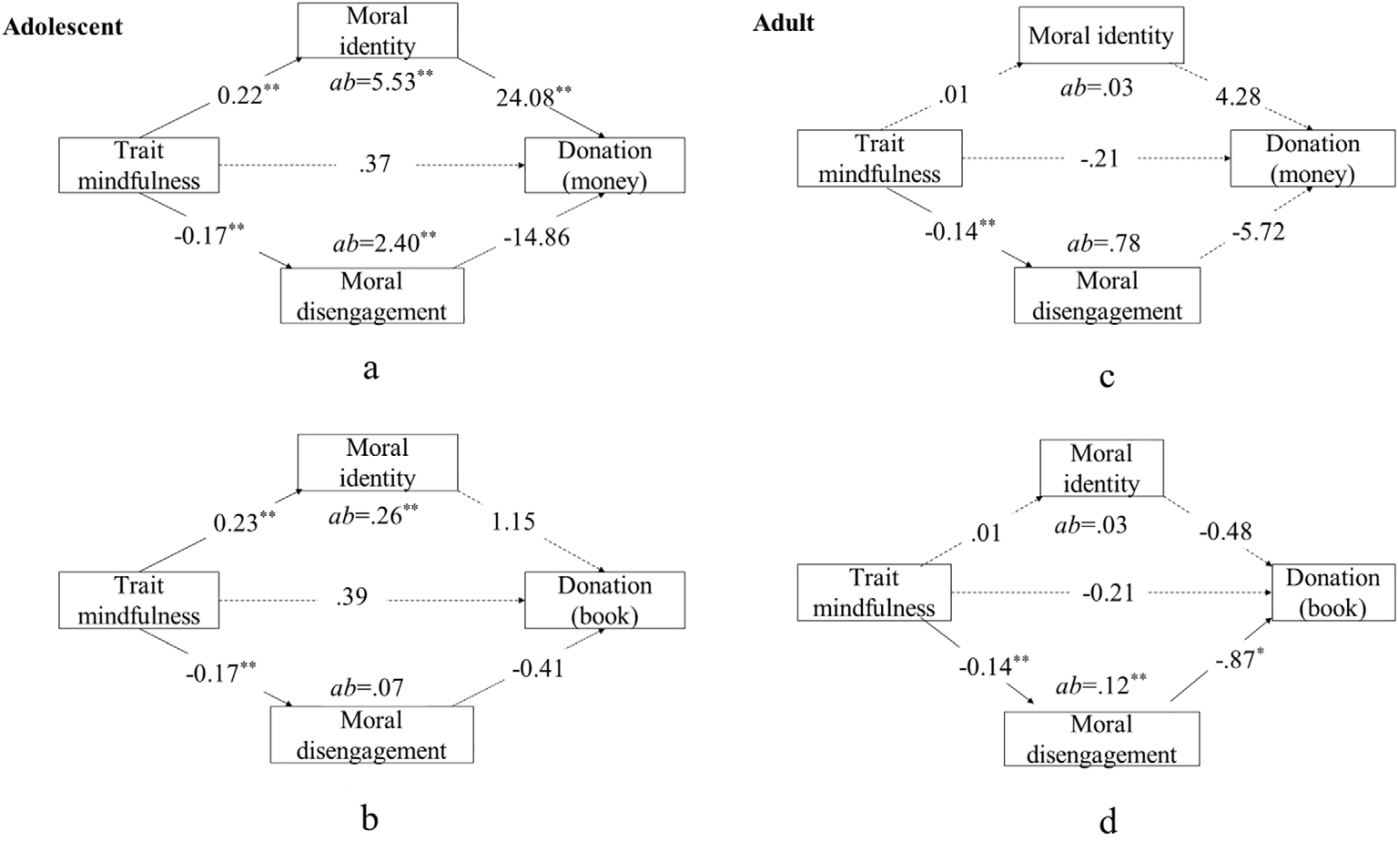
Mediation Model Graph for Young Adolescents and Young Adults, with Figures a and b referring to young adolescents and Figures c and d referencing young adults.

In the mediation analysis, dispositional mindfulness was designated as the independent variable, with MI and MD functioning as parallel mediating variables. The dependent variables included the willingness to donate both money and books. For the young adult group, one significant mediating effect was detected: MD significantly mediated the relationship between dispositional mindfulness and the willingness to donate books (*ab* = 0.12, SE = 0.07, CI [0.01, 0.29]; see Figure 1d). However, the mediating effect of MD between dispositional mindfulness and the willingness to donate money was not significant, nor were the mediating effects of MI regarding either willingness to donate books or willingness to donate money. In contrast, for the young adolescent group, MI significantly mediated the relationship between dispositional mindfulness and the willingness to donate money (*ab* = 5.53, SE = 4.46, CI [0.04, 16.73]; see Figure 1a) as well as the willingness to donate books (*ab* = 0.26, SE = 0.17, CI [0.02, 0.67]; see Figure 1b). However, the mediating effect of MD was found to be non‐significant for both the willingness to donate books and money (see Figure 1c,d).

### 2.6 Discussion

Our findings suggested that MI mediated the relationship between dispositional mindfulness and prosocial willingness in young adolescents, whereas MD played a more prominent role in young adults. These results highlighted the importance of age‐related differences. Conversely, the study did not reveal a mediating role for MD in the relationship between dispositional mindfulness and donation intentions. In the case of young adults, the findings indicated only a mediating effect of MD on the relationship between dispositional mindfulness and the willingness to donate books.

Nonetheless, these findings warrant further experimental validation. Prior studies indicated that brief mindfulness interventions or manipulations, such as approximately 5 min of mindfulness practice, could significantly enhance participants' willingness to contribute to charitable causes (Iwamoto et al. 2020). In this context, Study 2 is designed to extend the findings of Study 1 through a single short‐term mindfulness induction task. Additionally, Study 2 aims to investigate whether different forms of short‐term mindfulness induction—specifically secular mindfulness, which emphasizes non‐judgmental awareness, and ethical mindfulness, which incorporates ethical considerations—yield varying effects on young adolescents compared to young adults.

## 3 Study 2

### 3.1 Methods

#### 3.1.1 Participants

We recruited 105 undergraduate students (young adults) from a university in Chongqing through online announcements and randomly assigned them to one of three experimental groups: secular mindfulness, ethical mindfulness, or control. Four participants failed to complete the questionnaire, resulting in their data being deemed invalid and excluded from the final analysis. The final valid response rate was 96%, yielding a sample size of 101 participants (control group, *n* = 33; ethical mindfulness group, *n* = 31; secular mindfulness group, *n* = 37), with ages ranging from 19 to 23 years (*M*_age_ ± SD = 20.04 ± 2.36), and females comprising 56% (*n* = 57) of the sample.

The young adolescent sample consisted of 142 middle school students from 3 s‐grade classes at a junior high school in Chongqing, with each class randomly assigned to one of the three groups. Eight students were excluded due to incomplete or careless questionnaire responses, resulting in a final total of 134 participants (control group, *n* = 50; ethical mindfulness group, *n* = 41; secular mindfulness group, *n* = 43), with a mean age of 13 to 15 years (*M*_age_ ± SD = 13.46 ± 0.53), and 51% (*n* = 70) being female.

Based on the analysis of variance power calculations (effect size = 0.25, *α* = 0.05, power = 0.80), G*Power v.3.1 indicated a requisite sample size of 211 participants. Thus, the final sample size met this criterion.

### 3.2 Measures

#### 3.2.1 Moral Identity

The measures employed were identical to those used in Study 1. The Cronbach's alpha for the A‐MIQ in the present study was 0.92, while the Cronbach's alpha for the E‐MIQ was 0.61.

#### 3.2.2 Moral Disengagement Scale

The measures utilized were consistent with those in Study 1. The Cronbach's alpha for this scale among middle school students and college students in this study was 0.87 and 0.82, respectively.

#### 3.2.3 Hypothetical Donation Scenario

The measures were consistent with those employed in Study 1 and included two hypothetical scenarios regarding donations of money and books.

### 3.3 Procedure

The study was conducted at one middle school and one university in China. After obtaining informed consent, participants participated collectively in 25 min of either pre‐recorded secular mindfulness practice, ethical mindfulness practice, or relaxation practice within the laboratory setting. Subsequently, participants completed the MI scale, MD scale, and donation intention questions. Both pre‐recorded 25‐min mindfulness audio sessions comprised two parts: (a) mindfulness breathing and body awareness exercises (10 min) and (b) mindfulness writing tasks related to real or imagined prosocial events (15 min).

Mindful writing serves as an effective method for fostering awareness and enhancing one's understanding of reality (Medrano 2017). In the writing task, participants were instructed to recollect or imagine recent prosocial experiences, such as assisting others, while allowing relevant situations, imagery, thoughts, and emotions to surface uninhibitedly, maintaining a non‐judgmental attitude. They were also encouraged to remain fully aware of their current physical sensations, emotions, and thoughts, recording them honestly—whether perceived positively or negatively—while embracing them with an open mind.

For the ethical mindfulness induction task, drawing upon the ethical mindfulness meditation intervention described in Chen and Jordan's study (2018), we integrated principles such as “do not harm,” “all beings are interdependent,” and “all beings are equal” into the ethical mindfulness practice instructions, while also emphasizing physical and mental experiences and nurturing a non‐judgmental and accepting attitude. For instance, participants were guided with statements such as, “Please focus on your breathing… Recognize that the same air nourishes all people,” and “We are all equal. In that moment, consider that the happiness and pain of others are as significant as your own.”

The control group partook in 10 min of relaxation exercises, followed by 15 min of reflection on their past week, devoid of mindfulness‐related instructions. To maintain parity with the auditory material of the experimental groups, two instrumental music pieces were played for approximately 10 min for the control group.

### 3.4 Data Analyses

Data analysis was conducted using SPSS version 22.0. Initially, self‐reported scores for MI were aggregated into an overall mean score. Although different questionnaires were employed for college and middle school students, leading to varying total scores, the outcomes reflected comparable constructs, as all responses were assessed on a 5‐point scale. Subsequently, descriptive statistical analyses and analysis of variance (ANOVA) were performed on the data across the three groups. The PROCESS macro in SPSS was used to test the mediation models.

### 3.5 Results

Descriptive statistics and ANOVA were conducted to assess the effects of mindfulness induction (ethical vs. secular) on MI, MD, and prosocial willingness, as shown in Table 3.

**TABLE 3:** Descriptive statistics and analysis of ANOVA.

Participants	Group	Moral identity (*M* ± SD)	Moral disengagement (*M* ± SD)	Donation willingness (book) (*M* ± SD)	Donation willingness (money) (*M* ± SD)
Young adolescents	Control	3.37 ± 0.68	1.71 ± 0.31	8.17 ± 2.50	79.35 ± 24.81
	Secular mindfulness	3.85 ± 0.50	2.32 ± 0.43	8.05 ± 2.78	81.13 ± 27.30
	Ethical mindfulness	3.40 ± 0.57	2.01 ± 0.35	7.12 ± 3.07	63.96 ± 33.48
	*F* values	9.07***	33.28***	1.79 (*p* = 0.173)	4.37* (*p* = 0.015)
Young adults	Control	3.56 ± 0.60	2.15 ± 0.34	7.36 ± 3.20	75.73 ± 25.70
	Secular mindfulness	3.51 ± 0.49	2.18 ± 0.31	7.35 ± 2.65	72.57 ± 29.10
	Ethical mindfulness	3.64 ± 0.61	2.10 ± 0.38	6.94 ± 3.01	72.97 ± 26.90
	*F* values	0.44 (*p* = 0.65)	0.52 (*p* = 0.60)	0.22 (*p* = 0.80)	0.13 (*p* = 0.88)

*** *p* < 0.001.

* *p* < 0.05.

The ANOVA results revealed that the short‐term mindfulness‐based induction task had a significant effect on young adolescents' MI (*F*_(2,133)_ = 9.07, *p <* 0.001, *ɳ*_*p*_^2^ = 0.122), MD (*F*_(2,133)_ = 33.28, *p <* 0.001, *ɳ*_*p*_^2^ = 0.337), and willingness to donate money (*F*_(2,133)_ = 4.37, *p* = 0.015, Partial *ɳ* ^2^ = 0.066). Multiple comparisons showed that the MI self‐evaluation score for young adolescents in the secular mindfulness group was significantly higher than in both the control group (*p* = 0.001) and the ethical mindfulness group (*p* = 0.003). No significant difference was found between the ethical mindfulness group and the control group (see Table 3). The MD scores for young adolescents in both the ethical and secular mindfulness groups were significantly lower than those in the control group (*p* ≤ 0.001), with no significant difference observed between the ethical mindfulness group and the secular mindfulness group. Furthermore, the willingness to donate money for young adolescents in the ethical mindfulness group was significantly lower than that of the control group (*p* = 0.047) and the secular mindfulness group (*p* = 0.031), while no significant difference was found between the secular mindfulness group and the control group. However, about young adults, no significant differences were observed in MI, MD, or willingness to donate money or books.

**FIGURE 2:**
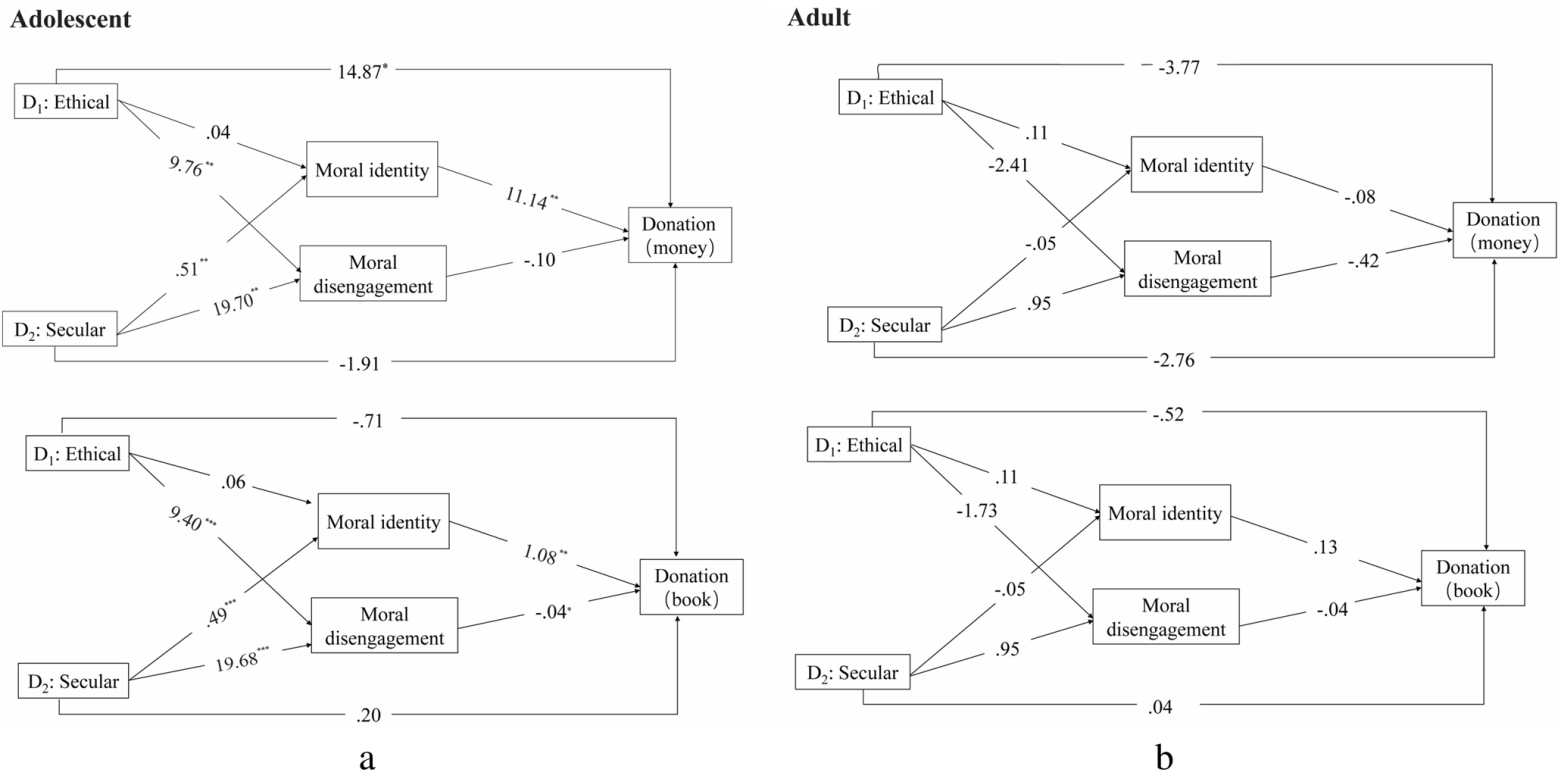
Mediation Analysis Model Plots for Different Mindfulness Induction Experiments, where Figure 2a refers to young adolescents and Figure 2b refers to young adults.

For the mediation analysis, the group variable was used as the independent variable (with the control group serving as the reference), while MI and MD were treated as parallel mediating variables, with the willingness to donate books and money serving as the dependent variables (see Table 4). The findings indicated that for young adolescents, the short‐term ethical mindfulness induction task had a significant mediating effect on the intention to donate books through MD compared to the control group (95% CI [−1.91, −0.03]). Additionally, the secular mindfulness induction task significantly influenced both book donation intentions (95% CI [−1.02, −0.01]) and money donation intentions (95% CI [1.73, 11.35]) via the mediating effect of MI. It also affected book donation intentions significantly through the mediating effect of MD (see Figure 2a). In contrast, for young adults, no significant mediating effects of MI or MD were found between the two mindfulness induction tasks and the willingness to donate money or books (see Figure 2b).

**TABLE 4:** The results of mediation analysis.

Independent	Meditator	a‐path					b‐path					Indirect effect			
		*B*	SE	*p*	CI		*B*	SE	*p*	CI		*β*	SE	CI	
					LL	UL				LL	UL			LL	UL
Dependent variable: money															
Participants: young adolescents															
MF	MI	0.22	0.04	< 0.001	0.14	0.32	24.08	8.26	< 0.001	7.79	40.36	5.53	4.46	0.04	16.73
	MD	−0.17	0.03	< 0.001	−0.23	−0.11	−14.03	12.09	0.24	−37.84	9.80	2.40	1.52	−0.81	5.27
Dependent variable: money															
Participants: young adults															
MF	MI	0.01	0.04	0.89	−0.08	0.09	4.28	5.53	0.44	−6.61	15.18	0.03	0.50	−1.01	1.17
	MD	−0.14	0.04	< 0.001	−0.22	−0.05	−5.72	5.19	0.27	−15.95	4.50	0.78	0.79	−0.51	2.61
Dependent variable: book															
Participants: young adolescents															
MF	MI	0.23	0.04	< 0.001	0.14	0.32	1.15	0.84	0.17	−0.50	2.79	0.26	0.17	0.02	0.67
	MD	−0.17	0.03	< 0.001	−0.23	−0.11	−0.41	1.22	0.74	−2.82	2.01	0.07	0.26	−0.54	0.47
Dependent variable: book															
Participants: young adults															
MF	MI	0.01	0.04	0.89	−0.08	0.09	−0.48	0.39	0.22	−1.25	0.29	−0.00	0.03	−0.08	0.08
	MD	−0.14	0.04	< 0.001	−0.22	−0.05	−0.87	0.37	0.02	−1.59	−0.14	0.12	0.07	0.01	0.29

Abbreviations: MD: Moral disengagement; MF, Mindfulness; MI, Moral identity.

### 3.6 Discussion

The results of Study 2 highlighted that young adolescents were particularly responsive to short‐term mindfulness inductions, with ethical mindfulness influencing MD and secular mindfulness affecting MI and prosocial willingness. In contrast, young adults showed minimal change, suggesting that interventions for adults might require a more sustained approach. The ANOVA results indicated that the secular mindfulness induction task positively influenced young adolescents, improving their self‐assessment scores for MI and willingness to donate money, while also increasing their self‐assessment scores for MD. Prior studies have indicated that mindfulness induction can improve cognitive performance in tasks involving episodic and autobiographical memory (Gill et al. 2020; Levi and Rosenstreich 2019). Consequently, compared to the control group, the mindful writing task, which prioritized non‐judgmental awareness, likely elicited a more pronounced positive emotional experience and facilitated enhanced recall of helpful scenarios, potentially leading participants to perceive themselves as more moral individuals. Contrary to our hypothesis, the ethical mindfulness induction task did not improve participants' MI scores or willingness to donate. Instead, it led to increased levels of self‐rated MD compared to the control group.

Furthermore, preliminary mediation analyses demonstrated distinct mechanisms by which the two mindfulness‐based induction tasks influenced young adolescents' intentions for prosocial behavior. Specifically, for young adolescents, the ethical mindfulness induction task primarily influenced the willingness to engage in prosocial behavior through MD, while the secular mindfulness induction task affected the willingness to participate in prosocial behaviors through both MI and MD. However, no mediating effects were identified among young adults. These findings highlighted the different relational models and psychological mechanisms by which MI and MD related to mindfulness and prosocial behavioral intentions for young adolescents and adults.

## 4 General Discussion

Overall, Studies 1 and 2 consistently supported our core hypothesis. When considering the relationship between mindfulness and prosocial willingness, or the effect of mindfulness intervention on prosocial willingness, the age‐related differences, such as the characteristics of MI and MD, need to be valued.

The results from Study 1 showed that young adolescents reported significantly higher total self‐rated MI scores, including the internalization and symbolization dimensions, compared to young adults. These discrepancies may reflect developmental differences in MI, with young adolescents often possessing more favorable perceptions of their moral competence than adults. Some researchers have suggested that, theoretically, mindful awareness could be related to moral cognition and behavior, as mindful awareness could be related to factors such as good self‐knowledge, awareness of moral situational cues, or good moral sensitivity (Sevinc and Lazar 2019). However, our findings suggested that the relationship between mindfulness orientation and prosociality was complex and could not be generalized. It is significantly influenced by age‐related traits of moral psychological development, such as MI, MD. Furthermore, this outcome may be linked to China's emphasis on moral education for primary and secondary school students. In China, moral education is considered a vital subject area alongside science and art, thus being prioritized within the curriculum. Primary and secondary school students are consistently guided to cultivate good moral consciousness. Such education emphasizes moral rules and principles; however, it often lacks grounding in social contexts (Chao 2021). Consequently, young adolescents in Chinese educational settings are encouraged to view the cultivation of their moral selves as a primary developmental objective. Conversely, young adults in Chinese universities exhibit significantly lower scores in the dimensions of internalization and symbolic MI while demonstrating significantly higher levels of MD in comparison to young adolescents. This phenomenon may result from evolving values and MI objectives characterized by commitment, flexibility, and openness to transformation (Schwartz et al. 2015). Furthermore, it may also stem from the relatively superficial and cursory approach to moral education prevalent at the university level in China (Wang and Zhang 2016), as well as the observable disconnection between the moral cognitions and behaviors of Chinese college students.

Analyses from Study 1 indicated that the relationships among dispositional mindfulness, MI, MD, and prosocial intentions were more pronounced among young adolescents compared to young adults. Such differences can be elucidated by the characteristics associated with individual moral self‐development and various influencing factors (Ding 2014). When an individual's moral self is deeply internalized, MI may not act as a positive mediator in the relationship between dispositional mindfulness and prosocial behavior. For instance, Poulin et al. (2021) found that brief mindfulness practice diminished prosocial inclinations in individuals with higher independent self‐construction when compared to the control group. Adults, who have internalized a relatively stable and consistent moral self and developed social cognition, often consider multiple factors—such as fairness (Hao et al. 2015)—when navigating moral dilemmas, rather than strictly adhering to external evaluative standards or striving solely to enhance their self‐image, as is more characteristic of young adolescents. Accordingly, individuals significantly anchored in their moral identities may regard beneficiaries as responsible for their predicaments and thus illustrate diminished benevolence, contrasting with individuals possessing lower levels of MI internalization. They may underscore the implications of MD and engage in heightened harmful behaviors when perceiving their counterparts as harboring fundamentally different moral or political values (Gotowiec 2019). This observation may partly elucidate why young adult students exhibited a positive correlation between MI and MD, in contrast to their adolescent counterparts. Additionally, this divergence may also relate to the instruments utilized for measuring mindfulness. Kil et al. (2021) identified that internalized prosocial motivation played a vital mediating role in linking mindfulness—specifically one dimension termed “acting with awareness”—to prosocial behavior. This observation underscores the necessity of investigating the specific effects of various mindfulness components on the relationship between mindfulness and prosociality. Overall, the most important result of study 1 is that MI and MD had significantly different associations between dispositional mindfulness and prosocial willingness for adolescents and young adults. This revealed the significant association of MI and MD between mindfulness and prosociality for young adolescents and provided insight into the moral mindfulness education of teenagers. That is, it is possible to improve prosociality by changing teenagers' MI and MD through mindfulness.

Study 2 confirmed the feasibility of this possibility. In other words, the moral psychology and behavior of adolescents are indeed more susceptible to external factors, including the short‐term mindfulness intervention in this study. The results of Study 2 also demonstrated that the short‐term secular mindfulness induction task had a more pronounced effect on young adolescents than ethical mindfulness tasks. For young adolescents, whose prefrontal cortex remains relatively immature (Crone and Dahl 2012) and whose self‐identity and values are undergoing rapid evolution, mindfulness induction or intervention emphasizing non‐judgmental awareness may produce unexpected repercussions on their social cognitive judgments and self‐awareness. Another possibility is that the non‐judgmental self‐awareness fostered during the training facilitated more authentic self‐perception (Zheng et al. 2020) and self‐acceptance, resulting in participants “seeing” various aspects of their true selves, which subsequently influenced their self‐ratings. Conversely, for young adults, who have transitioned into adulthood, neither short‐term mindfulness induction task appeared effective in influencing their self‐assessments of MI, MD, or prosocial willingness. During this developmental stage, young individuals seek to establish their uniqueness relative to others and cultivate new interests, values, objectives, and worldviews. Consequently, their behavioral judgments become less influenced by brief interventions.

In any case, these results highlighted the notion that the moral psychology and behaviors of young adolescents were more responsive to external influences. Overall, the significant implications of this study lie in revealing substantial differences in the associations between mindfulness and prosocial behavioral intentions among young adolescents and young adults. These findings bear considerable relevance for future research, as well as practical applications within clinical and educational contexts. One crucial aspect is recognizing that an intervention designed for adults may not be equally effective for younger adolescents due to the varying developmental stages of social cognition and emotions. This realization underscores the necessity for mindfulness intervention designs targeting younger adolescents to be aligned with their psychological development. Notably, many prior investigations into adolescent mindfulness interventions have failed to provide specific details of the employed programs or discuss any modifications made (Tan 2016). Directly adapting adult mindfulness intervention frameworks to young adolescents may not be advisable and could yield adverse effects. Developmental considerations and suitability must be accounted for when implementing mindfulness interventions across different groups, particularly among young adolescents or children. Additionally, the quality of mindfulness practice and the timing and duration of interventions are critical, often overlooked factors.

This study identified differences in the impacts of short‐term mindfulness‐based induction tasks on MI, MD, and willingness to donate between young adolescents and young adults. Nonetheless, the study is constrained by the lack of a long‐term mindfulness intervention and the absence of a pre‐ and post‐test comparison design. Meanwhile, in Study 2, the effectiveness of mindfulness induction was not evaluated. Moreover, as this is not a purely mindfulness induction practice, future studies could consider how to verify the effectiveness of short‐term mindfulness induction and benefit from investigating the observed effects through a long‐term randomized intervention experiment. Given the unique effects of mindfulness on younger adolescents, it is critical to account for interactive factors during mindfulness interventions tailored for this age group, particularly reflexivity, as posited by Etherington (2007). Reflexivity can facilitate ethical mindfulness by connecting self‐awareness with awareness of others and promoting relational consciousness. Finally, enhancing the ecological validity of studies examining prosociality—moving beyond merely hypothetical behavioral intentions—remains an area in need of further exploration.

## Ethics Statement

Ethics approval for this study was obtained from Zunyi Medical University. All procedures performed in the current study were by the ethical standards of the institutional and/or national research committee and with the 1964 Helsinki Declaration and its later amendments or comparable ethical standards. All authors state their compliance with the Code of Ethics of the World Medical Association (Declaration of Helsinki). The manuscript does not contain clinical studies or patient data.

## Consent

Informed consent was obtained from all individual participants included in the study. Informed consent clarified the voluntary, confidential, and anonymous nature of the study and data protection rights.

## Conflicts of Interest

The authors declare no conflicts of interest.

## Data Availability

The data that support the findings of this study are available upon reasonable request from the authors.
